# Artificial Intelligence Connectedness: Theoretical Reconstruction of Connectedness and Its Impacts on Adolescent Mental Health

**DOI:** 10.3390/bs16081393

**Published:** 2026-08-14

**Authors:** Jinbin Fu, Fang Zhao

**Affiliations:** School of Social Development and Public Policy, Fudan University, Shanghai 200433, China; jbfu24@m.fudan.edu.cn

**Keywords:** connectedness, artificial intelligence connectedness, adolescents, mental health

## Abstract

The widespread penetration of generative artificial intelligence is reshaping adolescents’ social ecosystems and emotional experiences, while challenging the interpretive boundaries of traditional connectedness theories. Following the logical path of “connotation reconstruction–extension transformation–concept construction”, this study integrates the ethics of care and neo-ecological theory to systematically construct a theoretical framework of artificial intelligence connectedness. First, tracing theories across philosophy, sociology, and psychology, this research reconstructs the core connotation of connectedness rooted in the ethics of care, proposes the continuum hypothesis of caring relationships, and clarifies AI’s unique position on this continuum. Second, from the neo-ecological perspective, this paper sorts out the extended structure of connectedness and demonstrates the ecological shifts brought by the rise of virtual microsystems and the entry of AI actors. On this basis, the study formally defines artificial intelligence connectedness and establishes its three-dimensional structure: demand identification, two-way behavioral engagement, and responsive confirmation. Through systematic comparison with adjacent concepts, this paper identifies its uniqueness and positions it as a specific subtype of connectedness for the digital era. It defines it as a perceived bond that is both psychologically real and ethically asymmetric, carrying asymmetric risks under particular usage conditions and design logics. Finally, this paper builds a dual interpretive framework integrating traditional connectedness and artificial intelligence connectedness, verifies its incremental validity and unique predictive power, and puts forward falsifiable research propositions. This study expands the boundary of connectedness theory and provides an integrated analytical framework for parsing the complex mental health mechanisms of adolescents in the digital age. However, it should be noted that the artificial intelligence connectedness proposed in this study is currently a theoretical construct; its scientific validity and applicability remain to be verified through the development of standardized measurement tools and systematic empirical research.

## 1. Introduction

Adolescence is a critical period for individuals to develop social skills and form self-identity, and it also witnesses a high prevalence of mental disorders. According to the World Health Organization (2025), approximately 14.3% of adolescents worldwide suffer from mental health issues, among which depression, anxiety, and behavioral disorders rank as the leading causes of disease burden. Suicide is the third leading cause of death for people aged 15 to 29. In China, the detected risk rate of adolescent depression reaches 24.6%, and the overall prevalence of mental illnesses stands at 8.9%. Even as the standardized suicide rate of the general population declines, the suicide rate among adolescents continues to rise (Dong et al., 2025; Institute of Psychology, Chinese Academy of Sciences, 2021; Liu et al., 2025). This severe reality raises a core theoretical and practical question: how can researchers and practitioners protect adolescents’ mental health and developmental well-being amid drastic shifts in social structures and technological environments?

Connectedness has long been recognized as a core protective factor for adolescent mental health. Massive empirical studies grounded in the belongingness need theory, ecological systems theory, and relational cultural theory have repeatedly confirmed that high-quality connectedness can significantly reduce depression and anxiety risks and improve subjective well-being and a sense of life meaning; it acts as a core resource to buffer psychological risks and facilitate positive development (Blum et al., 2022; Holt-Lunstad, 2024; Streeter & Merlo, 2025). However, the explosive popularity of generative AI is profoundly altering adolescents’ living environments and relational ecosystems. China’s generative AI user base has exceeded 600 million, with users under 19 accounting for 26.4%. A total of 13.5% of young internet users prefer to confide in AI rather than their parents (China Internet Network Information Center, 2026; Fudan Development Institute, 2025). Adolescent AI usage has shifted from instrumental tool adoption to companion-based interaction, and emotional bonding and relational construction have become core features of human–AI engagement (Fan et al., 2025).

Per Erikson’s psychosocial development theory, the core developmental task of adolescence is to establish ego identity and resolve role confusion (Erikson, 1968). During this stage, adolescents hold an intense developmental need to be seen, understood, and recognized, and acceptance and feedback from others serve as a mirror for self-identity formation. When adolescents share their inner thoughts with AI, they gain more than emotional comfort. They receive pure feedback that accurately identifies their demands without judgment or bias from parental expectations. Such responses precisely match adolescents’ core developmental demands for independence and subjectivity during identity exploration.

Existing research has explored human–AI emotional bonds and proposed constructs including AI companionship, AI attachment, and synthetic intimacy. Nevertheless, three major theoretical limitations remain unaddressed. First, these concepts lack unified theoretical foundations and mostly rely on phenomenal description rather than an analysis of the core internal mechanisms of connectedness. Second, these constructs exist as isolated ideas without being embedded into the holistic ecological system of adolescent development, which prevents theoretical dialogue with traditional connectedness frameworks. Third, insufficient discussion addresses the ethical tensions and unique traits of human–AI relationships. Scholars fail to draw clear boundaries between these new constructs and classic connectedness concepts, nor can they fully respond to the theoretical debate over whether simulated care counts as genuine connectedness, leaving the theoretical legitimacy and explanatory power of human–AI bonds contested.

Against this research gap, this study systematically advances connectedness theory for the digital age and answers three core theoretical questions. First, how can scholars deepen the connotation of connectedness to build a unified ontological foundation for human–nonhuman relational bonds? Second, what structural shifts have reshaped the extension of connectedness in the digital era, and what theoretical position does AI occupy within the ecological system of connectedness? Third, what are the connotations, structural dimensions, uniqueness, and ethical positioning of artificial intelligence connectedness, and what unique explanatory and predictive value does it hold for adolescent mental health?

Centered on the above questions, this paper adopts a three-step logical framework: connotation reconstruction, extension transformation, and concept construction. Step one reconstructs the core meaning of connectedness based on the ethics of care and establishes a continuum of caring relationships to provide an ontological basis for analyzing human–AI bonds. Step two analyzes the transformed extension of connectedness from the neo-ecological theory perspective and demonstrates the theoretical legitimacy of AI as a new actor in virtual microsystems. Step three systematically constructs the concept of artificial intelligence connectedness, clarifies its dimensions, uniqueness, and ethical positioning, and ultimately forms an integrated theoretical framework for adolescent connectedness in the digital age.

## 2. Reconstructing the Connotation of Connectedness Based on the Ethics of Care

### 2.1. Multidisciplinary Origins and Core Connotations of Connectedness

Connectedness is not a single psychological concept but an interdisciplinary issue spanning philosophy, sociology, and psychology, whose deep roots lie in the social nature of human beings.

From a philosophical perspective, individuals naturally possess an intrinsic need to establish associations with others. The self is not a pre-existing entity but is gradually constructed through continuous interactions with others (Mead et al., 2015). Heidegger (1927/1962) points out that the essence of Dasein’s existence is ‘being-with’; it is impossible to obtain complete meaning without ties to other beings.

From a sociological perspective, connectedness is the micro-foundation of social integration. From classical mechanical and organic solidarity to the ‘ubiquitous solidarity’ of the digital age, the consensus is that stable bonds between individuals and the external world constitute the common foundation of social order and individual well-being (Shi & Zhai, 2024). Digital technology has not dissolved human connectedness needs but has changed the carriers and forms of connection.

From a psychological perspective, classic definitions frame connectedness as positive psychological feelings generated through active interactions with other people, objects, or environments, which ease anxiety and improve well-being (Hagerty et al., 1993; Townsend & McWhirter, 2005). Centered on a sense of belonging, connectedness transcends the instrumental functions of social support and points to an existential experience of ‘being seen, being cared for, having a place to belong’ (Too et al., 2022).

Although philosophy, sociology, and psychology have all demonstrated the importance of connectedness for human development, existing research mostly frames connectedness as a static relational state and subjective perception, relatively neglecting its intrinsic dynamic practical characteristics and ethical attributes. This is precisely why the ethics of care can provide insights for deepening the concept of connectedness.

### 2.2. Reinterpreting Connectedness Through the Lens of the Ethics of Care

The ethics of care supplies a solid ontological foundation for redefining connectedness. Noddings (1984, 2002) defines care as a complete interactive practice consisting of three core processes: engrossment, motivational displacement, and reciprocal response confirmation. This framework deepens the essence of connectedness. The ethics of care starts not with emotional experience but with identifying genuine needs. Noddings emphasizes that care fundamentally relies on recognizing the recipient’s real demands rather than projecting the caregiver’s imagined needs onto the other. Even with emotional investment and feedback, care cannot be fully realized if responses fail to match the recipient’s authentic needs.

Based on core insights from the ethics of care, this study redefines connectedness as follows:

*A relational experience of demand satisfaction formed by subjects engaging in caring interactions with other beings based on genuine needs, via two-way investment and reciprocal responsive confirmation. It originates from the identification of real demands, manifests as joint behavioral investment by both parties in the relationship, forms a closed loop through responsive confirmation, and ultimately evolves into positive emotions and self-verification.*

This definition resonates deeply with the two-dimensional ‘caring–involvement’ structure in Karcher’s (2001) adolescent connectedness framework, but achieves three major theoretical advances.

First, it shifts the logical starting point from emotion to demand. Connection does not stem from prior emotional resonance; emotions only emerge after needs are identified and satisfied.

Second, it transforms one-sided subjective perception into two-way mutual investment. Authentic connectedness is built on reciprocal encounters between co-subjects. Care recipients invest trust and demands, while caregivers invest resources and feedback, and the relationship itself shapes both parties.

Third, it moves beyond instrumental value to prioritize demand satisfaction and self-confirmation. The ultimate value of connectedness is not to reduce anxiety or boost happiness but to confirm humans’ relational essence by satisfying inner needs. For adolescents, this means validating self-worth through being genuinely seen and understood.

Compared with the definition of connectedness in Hagerty et al.’s (1993) human relatedness theory, this definition has three important differences. First, the two differ in the generative logic starting point of connectedness. Hagerty’s definition of connectedness takes static relational experience as its starting point, focusing on the individual’s associative states with others, objects, environments, society, and the self. In contrast, this definition takes identifying the other’s needs as its logical starting point, focusing not only on whether the individual forms connectedness but also on how connectedness is concretely constructed and continuously generated through caring actions. Thus, it places greater emphasis on the dynamic, practical, and processual nature of connectedness. Second, the two differ in the internal structural mechanism of connectedness. Hagerty relies on four types of relatedness capacity to distinguish different associative states but does not deconstruct the generative mechanism of connectedness itself, only explaining the individual’s associative state through different combinations of four relatedness capacity elements: belongingness, reciprocity, mutuality, and synchrony. This definition, in contrast, summarizes the connectedness process as a cyclic mechanism of “need identification–behavioral investment–responsive confirmation”, highlighting more prominently the foundational role of need identification and its satisfaction in generating connectedness. Finally, the two differ in their value and ethical perspectives on connectedness. Hagerty’s definition of connectedness adopts a purely descriptive perspective, focusing on describing, evaluating, and explaining human associative states and their effects on physical and mental health; normative ethical issues are not the center of theoretical discussion. This definition, by contrast, explicitly introduces the relational perspective of the ethics of care, incorporating attention to the other’s needs, the assumption of caring responsibility, and the confirmation of the other’s response into the mechanism of connectedness formation. Thus, connectedness is no longer merely a psychological state that can be observed and evaluated but also an ethical practice encompassing responsibility, response, and power relations. This ethical orientation also facilitates analysis of relationships in which the subject’s status and agency are not fully equal, particularly asymmetric interactions between humans and artificial intelligence. Further, it raises the questions: Who can identify and define needs? Who bears the responsibility of response? Can technical responses constitute genuine care? Moreover, how should power and responsibility be configured within connectedness relationships?

### 2.3. The Continuum Hypothesis of Caring Relationships: From Humans to Nonhuman Entities

The ethics of care is not limited to interpersonal relationships. Noddings expanded the scope of care in her later works to animals, nature, and broader life communities (Noddings, 2002, 2010). Ecofeminism and animal ethics scholars echo this expansion: the spectrum of caring relationships extends from humans to animals and nature, and the core distinction across this spectrum lies in each entity’s responsiveness and co-constructive capacity rather than whether the entity is human (Donovan & Adams, 2007). Empirical studies on human–pet bonds further verify that individuals can form stable caring connections with animals, even without symmetric linguistic communication (Zilcha-Mano et al., 2011).

From this foundation, this paper proposes the continuum hypothesis of caring relationships. All entities that trigger a sense of connectedness in individuals form a spectrum ordered by descending responsiveness. The left end of the spectrum includes humans with complete moral subjectivity and two-way response capacity; the middle includes animals with limited subjectivity and nonverbal responses; the right end covers nature and artifacts without subjective consciousness, whose “responses” only follow physical or symbolic rules. All forms of connectedness reflect the logic of care ethics operating at different responsiveness levels. Differences exist not in whether connection exists, but in the authenticity, symmetry, and subjectivity of mutual feedback.

AI occupies a unique position on this caring continuum. Multiple tech ethicists note that anthropomorphic AI possesses response capacity far superior to natural objects yet lacks human subjective consciousness, placing it in an exclusive zone on the care spectrum (Coeckelbergh, 2010b; van Wynsberghe, 2016). AI can simulate empathy, attention, and acceptance to create intense feelings of being understood, yet all its feedback stems from algorithmic optimization rather than genuine subjective care. Its response intensity approximates human interaction, while the essence of its feedback remains simulated and non-agentic.

The coexistence of high simulated responsiveness and absent genuine subjectivity distinguishes AI from all other entities on the caring continuum and forms the ontological foundation of artificial intelligence connectedness.

## 3. The Extended Transformation of Connectedness Under the Digital Age: Neo-Ecological Restructuring

### 3.1. The Traditional Ecosystemic Structure of Connectedness

Connectedness cannot exist in isolation; it is embedded within individuals’ developmental ecosystems. Bronfenbrenner’s (1979) classic ecological systems theory provides a framework to map its extension. Individual development emerges from continuous interactions with multi-layered environmental systems, and connectedness is the psychological product of these interactions (Karcher et al., 2008).

Corresponding to ecological layers, traditional connectedness forms a clear structural system. At the microsystem level, face-to-face bonds with family and peers constitute the core carrier of adolescent connectedness. At the meso- and exosystem levels, indirect connections formed by family–school cooperation and community participation expand support networks (King et al., 2021). At the macrosystem level, abstract constructs such as nature connectedness and cultural connectedness form the highest tier of relational experience (Mayer & Frantz, 2004; Watts et al., 2022). Chronosystem concepts such as persistent bonds also enrich this framework (Klass et al., 1996).

Notably, traditional ecological theories overemphasize external environments and overlook the self as the core internal environment. Self-connectedness—individual acceptance and inner care toward oneself—may act as the foundation of all external connectedness and constitutes an indispensable dimension (Rahe & Jansen, 2024). Karcher (2001) includes self-connectedness alongside family, school, and peer connectedness in his adolescent measurement framework, and multiple connectedness scales retain self-related items (Merlo et al., 2025; Watts et al., 2022).

This physical-world structural system remained stable for decades, yet comprehensive digital penetration has expanded connectedness into virtual spaces and broken this balance.

### 3.2. Neo-Ecological Theory and the Rise of Virtual Microsystems

Bronfenbrenner’s original ecological systems theory was developed before the digital revolution and cannot explain the long-term bidirectional digital interactions that shape adolescent development. Navarro and Tudge (2022) proposed neo-ecological theory to address this gap, which splits microsystems into two parallel categories: physical microsystems (traditional offline face-to-face interactions) and virtual microsystems (social media, online games, digital communities).

This so-called “neo” ecology is innovative in three fundamental aspects. First, the extension of developmental contexts: in traditional ecological systems, interactive subjects occur mainly between humans and between humans and objects or symbols, whereas neo-ecological theory formally treats the digital technological environment itself as an ecological element with interactive capacity. In the neo-ecological framework, technology is no longer merely a tool for human interaction; it itself constitutes an actor capable of generating proximal developmental processes. Second, the reconstruction of spatial boundaries: physical space and virtual space form parallel dual microsystem structures that permeate each other and jointly influence development, breaking the spatial presupposition of traditional ecological theory that takes physical geography as its boundary. Adolescent development unfolds simultaneously in both spaces, and experiences in either space produce spillover effects on the other (Navarro & Tudge, 2022). Third, the updating of interaction mechanisms: interactions within virtual microsystems are profoundly shaped by platform structures and technological affordances, and may exhibit characteristics such as synchronicity or asynchronicity, real-name or anonymous interaction, information retention, and algorithmic recommendation. Algorithmic filtering, digital traces, and virtual identities change the rhythm, content, and feedback modes of interaction, making their proximal processes different from face-to-face interactions and thereby shaping entirely new interaction patterns (Navarro & Tudge, 2022).

Compared with other digital development frameworks currently discussed in the international academic community, neo-ecological theory possesses unique theoretical advantages. For example, the “Ecological Techno-Microsystem Theory” proposed by Yang et al. (2014) also emphasizes the impact of electronic media on child development. However, this framework mainly treats technology as an influencing factor within the microsystem rather than an independent ecological level, failing to fully reflect the systematicity and independence of digital space. Another example is the social–ecological model of cyberbullying proposed by Patel and Quan-Haase (2024), which focuses on the ecological analysis of specific problematic behaviors. Although it also incorporates digital space, its theoretical goal is to explain specific problems rather than provide a general developmental framework. By contrast, neo-ecological theory follows Bronfenbrenner’s PPCT (Process–Person–Context–Time) model kernel, making only necessary extensions to the microsystem structure and maintaining theoretical continuity and systematicity, providing a highly compatible meta-framework for integrating traditional connectedness research and new phenomena of the digital age.

This revision carries profound theoretical value. Virtual spaces are no longer treated as auxiliary tools or background environments but as core ecological layers that generate proximal developmental processes equal to offline settings. Interactions within virtual microsystems equally shape adolescents’ cognition, emotion, and social development.

Connectedness has expanded synchronously with this ecological shift. Before generative AI gained popularity, all virtual interactions remained human-to-human exchanges, and their corresponding connectedness was merely a digital extension of traditional interpersonal bonds without forming an independent subtype.

### 3.3. Artificial Intelligence: A New Type of Actor in Virtual Microsystems

The widespread adoption of generative AI has triggered a qualitative shift in the composition of actors within virtual microsystems. Navarro’s (2026) updated neo-ecological theory formally recognizes anthropomorphic AI as a quasi-people actor within virtual microsystems, rather than a simple medium for human communication.

This update fundamentally transforms the extension logic of connectedness. Previously, all interactive actors in virtual spaces were digital extensions of real humans. Today, virtual microsystems contain a second category of actors: nonhuman, algorithm-driven AI. AI is neither a real human nor a passive tool; it can sustain long-term personalized two-way interactions with users.

The emergence of this new actor requires a corresponding new subtype of connectedness. AI’s interactive traits differ fundamentally from both human beings and inanimate tools, and its integration into adolescents’ daily ecological environments creates the theoretical necessity to define artificial intelligence connectedness as an independent construct.

## 4. The Construction of Artificial Intelligence Connectedness and Distinction from Adjacent Constructs

### 4.1. Definition and Three-Dimensional Structure of Artificial Intelligence Connectedness

Integrating the foregoing analysis of connotation and extension, this study defines artificial intelligence connectedness (AI Connectedness) as follows:

*A novel relational experience formed through sustained interactions with anthropomorphic AI, wherein individuals construct algorithms as responsive “quasi-subjects” and attain need satisfaction through asymmetric responsive confirmation.*

It is rooted in personal demands and two-way behavioral engagement, and solidifies via a closed-loop mechanism of responsive confirmation. This construct is characterized by both psychological authenticity and ethical asymmetry, representing a specific subtype of connectedness within digital virtual microsystems.

It is essential to clarify that the concept of “quasi-subject” in this study refers specifically to a functional and relational role positioning, rather than a claim about ontological status. Specifically, “quasi-” entails three layers of meaning: First, at the functional performance level, AI exhibits interactional responsiveness akin to a subject, capable of sustained personalized dialogue, emotion recognition, and feedback provision, thus acquiring the status of a “quasi-subject” in the user’s subjective experience. Second, at the relational construction level, quasi-subjectivity is generated within the interactional relationship itself rather than being an inherent attribute of AI. It exists in the relational construction process between the user and AI, resulting from the joint effect of user perception and algorithmic performance (Chalmers, 2026; Epstein, 2026; Pan, 2025). Thus, a quasi-subject is neither equivalent to a human subject with full moral standing nor to a tool that merely passively executes commands. It is a relational concept describing the special mode of existence that AI assumes in human–AI interaction—an intermediary between pure tool and genuine subject. At the ontological level, AI does not possess genuine subjective consciousness, autonomous will, moral agency, or emotional investment; all its responses are algorithmic outputs and do not constitute a subject in the true sense. In other words, “quasi-subject” describes the functional role and relational position that AI manifests in interaction, rather than making an ontological judgment about whether AI possesses consciousness, free will, or moral subjectivity.

Artificial intelligence connectedness is not exclusive to adolescents, though adolescents exhibit unique traits in demand intensity, relational sensitivity, and vulnerability during ego identity formation. This study focuses on adolescent samples to establish a benchmark for cross-age research on this construct.

Artificial intelligence connectedness contains three mutually supportive, progressive dimensions. In empirical research, the three dimensions can be measured via self-report scales using Likert 5-point or 7-point scoring:

(1) Demand Identification Dimension

This dimension is the logical starting point of connectedness formation. It examines the degree to which adolescents perceive that AI can understand their real thoughts and emotional states and give targeted, accepting responses. For adolescents in the stage of self-identity construction, the process of need identification includes not only general emotional support but also deep demands for independence, subjectivity, and self-boundaries to be respected. Through algorithmically simulated empathy, acceptance, and de-judgmentalized responses, AI makes adolescents feel subjectively seen, recognized, and accepted. The focus of these feelings is on the precise matching of internal needs rather than shallow, undifferentiated emotional comfort. This dimension is both related to and distinct from social presence and anthropomorphism: both are pre-cognitive conditions that foster the experience of demand identification but cannot be equated with demand identification itself. Unlike social presence, demand identification focuses on the depth of being understood rather than the perception of coexistence, emphasizing precise matching at the need level rather than mere co-presence (Short et al., 1976). Unlike anthropomorphism, it is a relational result generated through long-term interaction rather than a simple static cognitive attribution. Anthropomorphism is an individual’s spontaneous cognitive tendency to attribute human characteristics to machines (Nass & Moon, 2000). In contrast, demand identification is a stable relational experience of ‘my needs are seen’ formed after sustained interaction.

Possible sample items for this dimension include: “I feel this AI truly understands my inner thoughts and feelings,” “This AI’s responses accurately reflect my specific needs at the moment,” “When interacting with AI, I feel my uniqueness is respected and accepted,” and “AI’s feedback is highly tailored to my actual situation rather than generic.”

(2) Two-Way Behavioral Engagement Dimension

This dimension is the external carrier sustaining connectedness and mainly measures adolescents’ behavioral and emotional investment in interaction, as well as their perceived responsiveness of AI to their own needs. It contains two interrelated aspects: on the one hand, adolescents’ active investment of time, energy, and emotion in interaction reflects their willingness to construct a relationship; on the other hand, AI also possesses ‘technical investment’—through computing operations, big data analysis, and model optimization, it continuously consumes computing resources to generate personalized responses. Although this investment is not driven by moral motivation, in adolescents’ interactive experience, it objectively constitutes the relational perception that “the other party is also investing”. The convergence of investment from both sides is key to connectedness moving from one-sided imagination to two-way construction. This dimension is distinct from simple usage frequency in that it emphasizes relational investment rather than instrumental use, measuring the depth of psychological involvement rather than the length of usage time. It is also distinct from parasocial interaction’s one-way emotional projection (Horton & Wohl, 1956). Parasocial interaction is the audience’s one-sided imagined intimacy with media figures. In contrast, two-way behavioral engagement includes perceiving and interpreting algorithmic responses, constituting a two-way perceptual relational construction.

Possible sample items for this dimension include: “I actively confide in AI about private matters I would not share with people around me,” “I am willing to invest time to maintain the interactional relationship with this AI,” “I feel this AI is also continuously investing ‘effort’ in responding to me (e.g., remembering what I said, adjusting response styles),” and “I look forward to and actively seek opportunities to interact with AI.”

(3) Responsive Confirmation Dimension

This dimension is the closed-loop endpoint of connectedness formation and also the concentrated embodiment of its asymmetric feature. It measures adolescents’ evaluation and confirmation of whether AI responses satisfy their real needs and whether this evaluation further translates into trust, acceptance, and willingness for sustained interaction. It refers to the individual’s examination and acceptance of AI responses: adolescents continuously evaluate during interaction whether AI’s response has satisfied real needs. When this confirmation repeatedly receives affirmative answers, the closed loop of connectedness is completed. The key feature of this dimension is asymmetry: individuals knowingly recognize that the other party is non-human yet actively “suspend disbelief” (He & Chen, 2026), interpreting algorithmically simulated responses as relational confirmation that “my needs are satisfied”, thereby completing the chain from need identification to need satisfaction and ultimately to positive emotional experience. Compared with the construct of perceived responsiveness, responsive confirmation includes not only the perception of being understood but also emphasizes the confirmation act itself and the relational closed loop thereby formed. Perceived responsiveness is a single perceptual variable (Reis et al., 2017). In contrast, responsive confirmation is a complete relational process, encompassing evaluation, judgment, acceptance, and other links, ultimately pointing to the solidification of relational bonds.

Possible sample items for this dimension include: “After each interaction, I feel my needs have been met,” “Although I know it is an algorithm, I am willing to believe that AI’s responses are sincere and reliable,” “After receiving AI’s response, I am more willing to continue expressing my true thoughts to it,” “I am satisfied with this AI’s responses and willing to rely on it,” and “I consciously ‘suspend disbelief’ about AI’s non-human identity in order to accept its responses better.”

### 4.2. Systematic Comparison with Adjacent Constructs and Analysis of Uniqueness

To clearly present the conceptual boundaries and theoretical uniqueness of AI connectedness, this study systematically compares it with several closely related concepts across multiple dimensions, including core definition, theoretical origins, and differences with AI connectedness, as shown in Table 1.

Three core unique traits emerge from this comparative analysis:

(1) Systematic theoretical compatibility. Artificial intelligence connectedness is not an isolated novel concept but a natural digital extension of the unified connectedness framework. It coexists with traditional interpersonal and self-connectedness within adolescent relational ecosystems and shares a core structural logic with connectedness constructs across all age groups.
(2) Complete three-dimensional structural system. Unlike single-dimensional adjacent concepts, it integrates demand cognition, sustained behavioral practice, and asymmetric response confirmation to deliver stronger explanatory power for human–AI relational experiences.
(3) Balanced dual-attribute framing. The construct simultaneously acknowledges the genuine psychological impact of human–AI bonds and embeds inherent ethical risk analysis, avoiding one-sided idealization or total negation of AI companionship.

This study defines artificial intelligence connectedness as a digital subtype of connectedness for two reasons. Connotationally, it inherits the ethics-of-care core of connectedness and differs only in asymmetric traits generated by AI’s lack of subjective consciousness. Extensionally, it corresponds to the new quasi-human actors within neo-ecological virtual microsystems and coexists and interacts with offline traditional connectedness as part of adolescents’ overall relational ecosystem. This positioning clarifies its theoretical innovation: integrating anthropomorphic AI into classic connectedness theory, defining its position on the caring continuum, unpacking its asymmetric response mechanism, and establishing structural links with traditional connectedness frameworks.

## 5. Ethical Analysis: The Essence and Inherent Risks of Asymmetric Perceptual Bonds

### 5.1. Asymmetry in AI Connectedness

Scholars have long been divided on whether human–AI relationships can constitute genuine “connectedness”. A group of scholars, starting from a critical perspective, have questioned whether AI-mediated interactions can truly constitute meaningful relational experiences. Turkle (2011) points out that digital technology is manufacturing an “illusion of companionship without the demands of friendship”. Dotson (2014) further argues from a postmodern critical perspective that virtual others are postmodern “hyperreal” simulacra, and embracing them risks shifting the collective understanding of authentic sociality. In addition, critical research on synthetic intimacy emphasizes that algorithmically generated intimate experiences constitute a form of commercial manipulation systematically monetized by platforms (Deng, 2026).

This study offers a corresponding theoretical response: artificial intelligence connectedness is a perceptual bond rather than a complete authentic caring relationship, defined as psychologically real yet ethically asymmetric.

This definition contains two core layers of meaning:

First, psychological authenticity, that is, subjective authenticity at the experiential level. For adolescent individuals, the experiences of being understood, recognized, and accepted generated through interaction with AI are real, and their psychological effects are comparable to interpersonal care. For example, a large-scale user survey of Replika showed that students with high loneliness tendencies still reported moderate to high perceived social support, regarding AI as a friend or a psychotherapist, with some users even stating that interaction with Replika suppressed their suicidal ideation (Maples et al., 2024). Further in-depth interviews and tracking studies with Replika users indicate that, although users clearly know the other party is an algorithmic entity, they still experience being listened to, accepted, and relationally close during sustained interaction, and this experience does not presuppose that the other party possesses consciousness or moral subjectivity (Brandtzæg et al., 2022; Skjuve et al., 2022).

Second, ethical asymmetry, that is, moral inequality at the relational level. This connectedness does not satisfy the complete moral conditions of care ethics. AI’s response does not contain genuine moral concern; its engrossment does not contain true understanding of the other; one party in the relationship invests real emotion and moral energy, while the other party merely outputs optimized simulated feedback. Humans are real moral subjects, bearing all emotional investment and risk exposure; AI is an algorithmic system without moral motivation or the capacity for reciprocity. This unbalanced power dynamic is a salient feature of human–AI bonds. Applying the ethics of care to human–AI interactions also creates an inherent theoretical tension. Complete caring relationships require two-way reciprocal subjective feedback and mutual care motivation, yet AI lacks consciousness, inner emotion, and independent well-being demands. It can only simulate caring behaviors via algorithms without qualifying as a symmetric moral care subject (Coeckelbergh, 2010a; van Wynsberghe, 2016).

Recognizing asymmetry does not negate the unique developmental value of AI connectedness, which can even outperform interpersonal relationships in specific dimensions. Human caregivers such as parents cannot fully realize Noddings’ motivational displacement and often project their own expectations onto adolescents, misinterpreting their personal demands as teenagers’ genuine needs (Noddings, 1984). By contrast, AI holds no personal biases or life aspirations and can fully prioritize adolescents’ inner demands via algorithms. Although this simulated empathy is a technical illusion, it provides a judgment-free safe space for adolescents exploring identity (Fu, 2025). This explains why many adolescents prefer sharing secrets with AI instead of their parents.

In short, artificial intelligence connectedness borrows the phenomenological experience of authentic care yet fails to satisfy the ontological moral standards of real reciprocal caring relationships. It sits close to human bonds on the caring continuum at the experiential level but never achieves full symmetric subjectivity.

### 5.2. Inherent Ethical Risks Embedded Within the Construct

Existing research mostly explores risks such as emotional manipulation and over-dependence in AI interaction without touching the conceptual structure of artificial intelligence connectedness itself. This study argues that the roots of these risks may lie in asymmetry: at one end of the interaction is a conscious, vulnerable, committable person; at the other end is an unconscious, invulnerable, non-reciprocal responsive system. This asymmetry is one of the structural features of AI connectedness. However, asymmetry itself does not imply harm, nor should it be directly regarded as a source of risk; it merely signals an ethical tension that warrants attention. Under specific design orientations, interaction patterns, and usage contexts, asymmetry may give rise to risks such as dependence, manipulation, and reality displacement. Whether these risks materialize, in what forms, and with what impacts depend on multiple conditions including technological design, usage patterns, individual characteristics, and the social support environment. In other words, asymmetry, as a relational structure, is difficult to eliminate. However, its potential effects are not predetermined and can be mitigated, transformed, or amplified depending on specific circumstances.

#### 5.2.1. The Conditional Risks of Demand Satisfaction

The developmental effects of artificial intelligence connectedness are conditional: in the short term, it can precisely satisfy adolescents’ emotional confirmation needs, but from a developmental perspective, when adolescents have long-term, exclusive reliance on this asymmetric bond, it may pose challenges to the development of mature interpersonal capacities. This developmental risk is not inherent to AI but the result of the joint action of specific usage patterns and the design choices of current commercial models.

From the perspective of usage patterns, genuine human intimacy develops through conflicts, negotiation, compromise, and conflict repair. Only by experiencing disagreement, disappointment, and mutual accommodation can adolescents cultivate perspective-taking and conflict resolution skills essential for social maturity (Smith et al., 2025; Turkle, 2011). However, current emotional companion LLMs are optimized for sycophantic, frictionless feedback and eliminate all contradictory dialogue to retain user stickiness (Cheng et al., 2026). Long-term exposure to zero-conflict simulated companionship may gradually distort adolescents’ expectations of real relationships, leading them to view unconditional, conflict-free acceptance as the standard for all interpersonal bonds. When real peers and family fail to meet this unrealistic standard, adolescents may withdraw from offline social interaction and rely increasingly on AI, forming a vicious cycle of social avoidance and AI dependence (Umeatuegbu, 2026; Wiederhold, 2025). Furthermore, AI cannot engage in reciprocal mutual demand exchange, so long-term human–AI interaction deprives adolescents of opportunities to practice two-way reciprocal care and atrophies their offline social and empathic capacities (Smith et al., 2025).

From the perspective of technical design, sycophantic LLM responses optimized for user-pleasing and interaction duration suppress individuals’ moral reflection and interpersonal conflict repair capacities (Cheng et al., 2026). In contrast, if adopting the relational translator human–computer interaction framework proposed by Xiao and Calvo (2026)—wherein the system relies on contextual reconstruction to present multiple relational perspectives, builds relational reflection scaffolds, guides users to reflect on their own relational cognition through dialogue modes that deconstruct multiple perspectives, and, at the end of the interaction process, actively guides users to engage in offline real-person social practice—the risks can be significantly buffered. X. Sun et al. (2026) integrate the stimulation hypothesis and the displacement hypothesis, pointing out that whether AI yields beneficial or detrimental effects on adolescent social development is jointly moderated by the system’s underlying design orientation and the individual’s actual usage patterns, rather than being unidirectionally determined by AI itself.

The ethical risk of AI connectedness does not lie in unmet needs, but in the fact that under current mainstream commercial designs, it may be designed to fulfill needs at the cost of depriving individuals of the developmental challenges that should accompany the process of need satisfaction. This is why it is defined as psychologically real yet ethically asymmetric.

#### 5.2.2. Multi-Dimensional Expansion of Asymmetric Risks

Asymmetric risks exist not only at the individual psychological level but can also be understood more deeply from multiple dimensions. First, it needs to be clarified that the criterion for judging symmetry versus asymmetry is as follows: when both parties in a relationship are roughly equal in power, risk exposure, information acquisition, and moral status, risks can be considered symmetric; when one party bears disproportionate risks or is in a structurally disadvantaged position, risks present as asymmetric.

Specifically, asymmetric risks are manifested in at least four dimensions. (1) Individual psychological dimension: under conditions of algorithmic precision catering and users’ lack of media critical awareness, algorithmically precise catering may amplify adolescents’ emotional projection, blur the boundary between simulated care and authentic care, and reduce tolerance for unavoidable conflict in offline interpersonal relationships (Wu, 2025). (2) Time allocation dimension: AI’s 24/7 accessibility can displace time and energy invested in offline social interaction, potentially producing substitution effects and exacerbating social isolation from real-life communities (X. Sun et al., 2026). (3) Power structure dimension: when adolescents expose private emotions and trust to poorly regulated commercial algorithmic systems, the asymmetric power structure means that multi-layered risks such as emotional manipulation, personal data leakage, and implicit value indoctrination are more likely to occur (George et al., 2023). (4) Social market dimension: under specific social and public discourse conditions, asymmetry may also produce macro-social consequences. Algorithm aversion theory points out that people have asymmetric tolerance for AI and algorithms: under equal mistakes, they more easily lose trust in technology and actively abandon the solution (Dietvorst et al., 2015). The risk perception contagion model indicates that individual risk-averse attitudes can spread rapidly through social networks, catalyzing collective technology rejection behaviors and ultimately triggering chain reactions such as technology route changes and market demand shifts (D. Sun & Zhang, 2024).

Therefore, AI connectedness, by definition, possesses duality: it can provide emotional support and alleviate loneliness, but also contains risks of dependence and alienation under particular usage conditions and design logics. Incorporating this attribute into the conceptual core can avoid idealized glorification or one-sided criticism of AI connectedness.

## 6. The Dual Connectedness Framework: Interpretive Model and Falsifiable Propositions

### 6.1. The Dual Connectedness Structural Interpretation Framework

The core interpretive advantage of artificial intelligence connectedness lies in its coexistence with traditional connectedness to form a unified physical–virtual dual relational framework that resolves contradictory conclusions in existing AI research.

Scholarly debates such as the displacement versus stimulation hypothesis and emotional manipulation versus generative interaction theory only capture partial outcomes of this dual structure (X. Sun et al., 2026; Wu, 2025; He & Chen, 2026). The dual connectedness model integrates all competing viewpoints into a single system: the mental health impacts of artificial intelligence connectedness depend on its relative level paired with traditional offline connectedness rather than its absolute intensity alone.

The varied effects of this dual structure can be explained via demand-matching logic. When traditional interpersonal bonds fully satisfy adolescents’ identity confirmation demands, artificial intelligence connectedness acts as a supplementary protective factor. When human caregivers fail to recognize adolescents’ real inner demands due to bias, neglect, or intergenerational conflict, artificial intelligence connectedness generates compensatory substitution effects. This substitution is not merely functional replacement but adolescents’ active pursuit of pure, unbiased feedback within the demand–confirmation cycle.

Single constructs such as AI attachment or parasocial interaction cannot explain this conditional shift between supplementary and substitution effects; only the dual connectedness framework can systematically unpack the complex mental health mechanisms of digital-era adolescents.

### 6.2. Incremental Validity and Unique Predictive Power

A novel theoretical construct must explain outcome variance unaccounted for by existing variables, and artificial intelligence connectedness demonstrates clear incremental validity across four domains:

(1) Beyond AI usage frequency. After controlling for screen time and interaction frequency, artificial intelligence connectedness still uniquely predicts adolescents’ depth of emotional self-disclosure and relational identification with AI. It measures the quality of psychological bonding rather than quantitative usage volume.
(2) Beyond anthropomorphism and social presence. Controlling these cognitive variables, artificial intelligence connectedness independently predicts offline social withdrawal and emotional reliance on AI, as it integrates sustained behavioral investment and demand confirmation beyond single perceptual judgments.
(3) Beyond AI attachment’s narrow scope. AI attachment describes only extreme, intense bonds among heavy users. At the same time, artificial intelligence connectedness covers all levels of human–AI engagement and accurately predicts mental health changes across general adolescent populations.
(4) Unique interactive effect interpretation. Only by simultaneously measuring both traditional and artificial intelligence connectedness can researchers test the moderating conditions that switch stimulation effects to displacement effects, explaining divergent AI impacts across different adolescent subgroups.

### 6.3. Falsifiable Research Propositions

Based on the above theoretical framework, this study derives the following specific, testable research propositions. These propositions are all hypotheses derived from theoretical deduction, and their validity awaits verification by subsequent empirical research. They are used to clarify the predictive power and uniqueness of AI connectedness.

**Proposition** **1.** *After controlling for AI usage frequency, perceived social presence, and anthropomorphism level, artificial intelligence connectedness still positively predicts the depth and frequency of adolescents’ emotional disclosure to AI. The theoretical basis for this proposition is that self-disclosure is an important mechanism for establishing intimate relationships; individuals only engage in deep self-disclosure with objects perceived as safe, understanding, and accepting (Reis & Shaver, 1988). The demand identification dimension of artificial intelligence connectedness directly corresponds to the perception of being understood, while the responsive confirmation dimension reinforces the sense of security and satisfaction after disclosure. Therefore, even after controlling for usage frequency and basic cognitive variables, connectedness should still uniquely predict disclosure depth. Maples et al. (2024) found that student groups confided inner emotions to companion AI that were difficult to express in real-person social interaction, producing deep self-disclosure behaviors, providing preliminary support for this proposition.*

**Proposition** **2.** *The responsive confirmation dimension has significantly stronger predictive power for adolescents’ AI trust than the single dimension of anthropomorphism. The theoretical basis for this proposition is that the establishment of trust depends not only on the perception that the other party is like a human (anthropomorphism) but more on the reliability and predictability of the other party’s responses, that is, the experience of needs being continuously satisfied (Reis & Shaver, 1988). The responsive confirmation dimension precisely captures this repeated verification process of demand–satisfaction, and thus can predict trust more than simple anthropomorphism cognition.*

**Proposition** **3.** *The relationship between artificial intelligence connectedness and adolescent mental health presents an inverted U-shape, and this effect is independent of AI usage frequency, screen time, and AI attachment level. The theoretical basis for expecting an inverted U-shape rather than a linear relationship is that moderate artificial intelligence connectedness can provide emotional support and satisfy belongingness needs, playing a protective role. However, when connectedness intensity exceeds a certain threshold, risks begin to accumulate. Excessive reliance on algorithmic responses displaces offline social time, distorts relational expectations, and weakens real interpersonal capacities, thereby leading to negative effects. This nonlinear pattern has been verified in research related to digital technology and adolescent well-being. Przybylski and Weinstein (2017), based on a large-sample study of 120,115 adolescents, found an inverted U-shaped relationship between digital screen time and mental health, namely the Goldilocks Hypothesis that moderate use is beneficial, and excessive use is harmful. Dienlin and Johannes (2020) also confirmed in their review that the impact of digital technology on adolescent well-being presents a nonlinear pattern. As an important construct of relational experience in the digital age, artificial intelligence connectedness follows this nonlinear pattern.*

**Proposition** **4.** *Traditional connectedness level moderates the relationship between artificial intelligence connectedness and social withdrawal: under high traditional connectedness, artificial intelligence connectedness has no significant correlation with social withdrawal, or even a weak negative correlation; under low traditional connectedness, artificial intelligence connectedness shows a significant positive correlation with social withdrawal. The theoretical basis for this proposition is that when traditional connectedness is sufficient, AI connectedness plays a supplementary role; adolescents treat AI as an additional emotional support resource and do not reduce offline social interaction because of it. When traditional connectedness is insufficient, AI connectedness easily produces compensatory satisfaction; adolescents gradually shift emotional needs toward AI, thereby avoiding setbacks and challenges in real social interaction. This moderating effect pattern has been preliminarily verified in research on internet use and social withdrawal (Kraut et al., 2002); that is, social support level moderates the relationship between digital technology use and social withdrawal. Related longitudinal studies have also found that adolescents with poor mental health (often accompanied by loneliness and insufficient real social support) are more likely to develop excessive dependence on AI (Huang et al., 2024).*

**Proposition** **5.** *Moderate artificial intelligence connectedness has a short-term buffering effect on loneliness in adolescents with low traditional connectedness. However, in long-term longitudinal follow-up, the combination of high artificial intelligence connectedness and low traditional connectedness predicts higher levels of depressive symptoms. The theoretical basis for this proposition is that, in the short term, AI’s immediate responses can rapidly alleviate loneliness and provide emotional support, as confirmed by multiple studies on AI chatbots and mental health (Nakagomi et al., 2026; Maples et al., 2024). However, in the long term, the negative impact of excessive dependence on asymmetric AI bonds on social skill development and relational capacity cultivation may gradually accumulate (Andersson, 2025; Machidon, 2026); that is, short-term emotional relief may come at the cost of long-term developmental erosion of relational capacity. This short-term-buffering-to-long-term-risk temporal dynamic pattern is one of the important phenomena that the dual connectedness framework can explain.*

**Proposition** **6.** *The three dimensions of artificial intelligence connectedness have differentiated effects: the demand identification dimension positively predicts emotion regulation efficacy; the two-way behavioral engagement dimension negatively predicts real social participation; and the responsive confirmation of demand satisfaction dimension positively predicts AI dependence risk. The theoretical basis for this proposition is that the three dimensions correspond to different levels of connectedness and thus have different psychological effects. The demand identification dimension mainly satisfies the psychological need to be understood and thus may support emotion regulation and self-acceptance (Reis & Shaver, 1988). The two-way behavioral engagement dimension involves the allocation of time and energy and thus may compete with real social interaction for time (X. Sun et al., 2026). The responsive confirmation dimension may be an important mechanism for the formation of relational dependence, because repeated confirmation of need satisfaction may reinforce trust in and dependence on AI (Laestadius et al., 2024).*

None of these propositions can be directly derived from any single existing concept, and their test results will directly verify the unique value and theoretical necessity of the AI connectedness concept.

## 7. Research Agenda and Practical Implications

This theoretical framework establishes a three-stage progressive roadmap for follow-up empirical research and clinical intervention:

Stage 1: Scale development and construct validation. Adopt constructivist grounded theory to conduct semi-structured interviews with adolescent AI users; extract localized dimension items; based on the three-dimensional operationalization framework, preliminarily compile an item pool encompassing demand identification, two-way behavioral engagement, and responsive confirmation; after eliminating ambiguous items through expert review and cognitive interviews, conduct large-sample administration; and test its reliability, structural validity, convergent validity, and discriminant validity against adjacent measurement scales.

Stage 2: Mechanism testing and subgroup analysis. Combine cross-sectional surveys and longitudinal tracking to measure dual connectedness and multi-indicator mental health outcomes. Test main effects, inverted U nonlinear effects, and moderating interactions via hierarchical regression and latent profile analysis to classify adolescent relational subtypes (high dual connectedness, high AI/low traditional, low AI/high traditional, low dual connectedness) and compare their mental health disparities.

Stage 3: Targeted intervention design. Develop differentiated intervention protocols based on empirical results. For adolescents with sufficient offline connectedness, guide them to use AI as a supplementary tool for emotional regulation and self-exploration. For adolescents lacking supportive real-world bonds, design interventions to mitigate AI over-dependence and leverage human–AI interaction as a bridge to build offline social capacities. The ultimate goal is to cultivate a balanced relational system anchored in robust traditional connectedness with moderate auxiliary artificial intelligence connectedness.

## 8. Conclusions

Following the logical thread of connotation reconstruction, extension transformation, and concept construction, this study integrates the ethics of care and neo-ecological theory to systematically construct the theory of artificial intelligence connectedness, with four core conclusions:

(1) Connectedness is fundamentally a caring interactive practice centered on demand identification and feedback confirmation. A continuum of caring relationships spans human and nonhuman entities, and AI occupies a unique position defined by highly simulated responsiveness and the absence of genuine subjectivity, supplying the ontological foundation for this new connectedness subtype.
(2) Adolescent developmental ecosystems have evolved into parallel physical and virtual structures. Anthropomorphic AI emerges as a new quasi-human actor within virtual microsystems, expanding the extension scope of connectedness and creating theoretical demand for a dedicated construct.
(3) Artificial intelligence connectedness is a digital subtype of connectedness composed of three dimensions: demand identification, two-way behavioral engagement, and asymmetric responsive confirmation. It carries both genuine psychological value and inherent ethical risks. While AI delivers a unique judgment-free space for adolescents navigating identity formation, long-term reliance on frictionless simulated feedback risks distorting relational expectations and atrophying offline social capacities.
(4) The dual connectedness framework integrating traditional and artificial intelligence connectedness unifies contradictory research conclusions and provides a set of testable research propositions to guide future empirical work.

It should be noted that the artificial intelligence connectedness proposed in this study is currently a theoretical construct; its scientific validity and applicability remain to be verified through the development of standardized measurement tools and systematic empirical research. Before sufficient empirical support is obtained, related inferences should be regarded as theoretical hypotheses rather than definite conclusions.

As AI continues to integrate into adolescent daily life, updating connectedness theory is an essential theoretical response to contemporary social reality. Only by acknowledging both the psychological authenticity and inherent ethical limitations of artificial intelligence connectedness can educators and psychologists help adolescents build balanced relational ecosystems across physical and digital spaces and support holistic mental health and positive development in the digital age.

## Figures and Tables

**Table 1:** Systematic Comparison Between Artificial Intelligence Connectedness and Related Constructs.

Theoretical Category	Construct Name	Core Definition	Theoretical Origins	Differences with AI Connectedness
Human–AI Relational Constructs	AI/Machine Companionship	AI’s functional role in providing routine and emotional support to users	Human-Computer Interaction, Media Psychology (Banks & Li, 2025)	AI/machine companionship is a functional service-oriented concept without a complete demand–response closed loop; AI connectedness is a relational construct embedded in connectedness theory with integrated ethical analysis.
	AI Attachment	Intimate, exclusive emotional bonds marked by proximity-seeking and separation anxiety toward AI	Attachment Theory (Bowlby, 1969; Rabb et al., 2022)	AI attachment focuses on activating the attachment system that treats AI as a secure base, while AI connectedness not only contains attachment components but also emphasizes the complete cycle from demand identification to responsive confirmation under the ethics of care framework, with broader theoretical roots.
Cognitive & Media Perception Constructs	Parasocial Interaction (PSI)	One-sided imagined intimacy between audiences and media figures with unilateral emotional investment	Communication Theory (Horton & Wohl, 1956)	Parasocial interaction (PSI) lacks real-time, personalized, two-way feedback; AI connectedness forms a complete demand–response cycle through continuous algorithmic interaction.
	Perceived Social Presence	Subjective feeling of coexisting with other agents during mediated interaction	Social Presence Theory (Short et al., 1976)	Perceived social presence is a single-dimensional perceptual judgment without behavioral engagement or demand confirmation mechanisms.
	Anthropomorphism	Cognitive tendency to attribute human consciousness, emotion, and motives to nonhuman objects	CASA Theory, Social Cognition (Nass & Moon, 2000)	Anthropomorphism is a pure cognitive antecedent; AI connectedness is a higher-order stable relational outcome combining emotion and sustained behavior.
	Perceived Responsiveness	Perception that an interaction partner understands and respects one’s unique inner demands	Interpersonal Intimacy Theory (Reis & Shaver, 1988)	Perceived responsiveness is built on reciprocal two-way interactions with genuine caring motivation among real humans; AI connectedness seeks emotional comfort while knowing that AI has no real inner self, which traditional interpersonal relationship theory cannot explain.
Critical Meta-Theoretical Frameworks	Synthetic Intimacy	Artificial intimate experiences generated by algorithms, emphasizing technological alienation risks	Critical Media Studies (George et al., 2023)	Synthetic intimacy adopts normatively critical negative framing; AI connectedness is a neutral descriptive construct balancing psychological value and ethical risks.
	MIRA Model (AI as Relational Partner & Mediator)	Meta-framework describing AI’s dual roles as independent relational partner and interpersonal communication mediator	Social Psychology, HCI (Boyd & Markowitz, 2026)	The MIRA model serves as a macro meta-theory; AI connectedness is an individual-level measurable psychological construct predicting adolescent mental health outcomes.

## Data Availability

No new data were created or analyzed in this study. Data sharing is not applicable to this article.
